# Feasibility and physical health outcomes of a 1-year sport-based physical activity intervention in rural middle school children

**DOI:** 10.1186/s13102-025-01498-4

**Published:** 2026-01-03

**Authors:** Sydney H. Callahan, Julianna F. King, Bailey K. Ortyl, Noah G. Cumming, Janette M. Watkins, Janelle M. Goss, Vanessa M. Martinez Kercher, Kyle A. Kercher

**Affiliations:** 1 Department of Kinesiology, School of Public Health-Bloomington, Indiana University, Bloomington, IN USA; 2 Department of Applied Health Science, School of Public Health- Bloomington, Indiana University, Bloomington, IN USA; 3 Department of Epidemiology and Biostatistics, School of Public Health- Bloomington, Indiana University, Bloomington, IN USA; 4 Department of Health & Wellness Design, School of Public Health- Bloomington, Indiana University, Bloomington, IN USA

**Keywords:** Service-learning, School-based intervention, Positive youth development, Sport-based youth development, Sport participation

## Abstract

**Background/Objective:**

Rural children face challenges to participating in physical activity (PA). These barriers include lack of facilities, adequate equipment, trained personnel, and transportation limitations. The primary objective of this study was to evaluate intervention feasibility, particularly focusing on the trial- and intervention-related indicators. The secondary objective was to assess preliminary efficacy and outcome trends in accelerometer-collected PA data from pre-, mid-, to post-intervention, as well as changes in physical measures (i.e., maximum plank test, 6-minute walk test).

**Methods:**

We conducted a 1-year controlled cohort study of 83 6th-8th grade children attending an under-resourced rural middle school in the Midwestern United States. The intervention, Hoosier Sport, was implemented by trained college students in an undergraduate service-learning course. The test group participated in enhanced PE classes including various sports and positive youth development lessons. The control group participated in standard health class curriculum. The primary outcomes were trial- and intervention-related feasibility indicators for children and college student implementers. The measures included AIM (Acceptability of Intervention Measure), IAM (Intervention Appropriateness Measure), and FIM (Feasibility of Intervention Measure), as well as recruitment and retention. Secondary outcomes included light, moderate, vigorous, and total PA, self-reported weekly PA, a maximum plank test, and 6-minute walk test. Given the repeated measures design at three time points (pre-, mid-, and post-intervention) and our primary interest in detecting within-subject changes and between-group differences, analysis focused on linear mixed models with within–between interaction effects.

**Results:**

FIM (median = 17), AIM (median = 18), and IAM (median = 17) scores exceeded the success threshold (median > 16) when assessed at post-intervention. Total minutes of PA decreased from baseline to mid (*β* = -42.762, 95% *CI*(-101.220, 15.696)) and baseline to post (*β* = -35.952, 95% *CI*(-89.617, 17.713)), but neither difference was significant (*p* = 0.149 & 0.185, respectively). At baseline, the test group estimated 64.91 more total minutes of PA than the control group (*t*(101.13) = 1.82, *p* = 0.072, *dz* = 0.771), with a marginally significant difference. There were no significant differences in change over time of total or self-reported PA between the groups (*p* > 0.05).

**Conclusions:**

The 1-year intervention was feasible to implement, with high appropriateness and acceptability among rural middle school children. Refinements are needed to continue targeting improvements in children’s PA.

**Trial registration:**

ClinicalTrials.gov ID: NCT06602596 Registration Date: 09.17.2024.

## Introduction

Cardiovascular disease (CVD) is the leading cause of death for both men and women in in the US, with a disproportionate impact on individuals in rural areas and those of lower socioeconomic status (SES) [1]. However, several modifiable risk factors can be addressed through lifestyle and behavioral changes to reduce the risk of CVD. Integrating regular physical activity (PA) throughout childhood increases the likelihood of maintaining an active lifestyle into adulthood. Conversely, a sedentary lifestyle contributes to increased risk of CVD and obesity [2]. Despite current guidelines for children’s PA, only 20–28% of children ages 6 to 17 engage in the advised 60 min of daily PA [3], and PA levels may continue to decline with age [4].

The decline in PA beginning in childhood adversely affects individuals in rural, low socioeconomic communities [5]. Disparities in PA often stem from social determinants of health, including economic stability, neighborhood environment, and social context [6]. Specifically, in rural communities where lower SES and inadequate infrastructure can limit access to transportation, extracurricular activities, and access to parks, playgrounds, and sidewalks [7]. These barriers impact families, ultimately limiting children’s ability to create and maintain physically active lifestyles. Low SES may also restrict parental involvement as supporters or role models for PA due to work obligations and time constraints, limiting their ability to attend or provide transportation for practices and events [6], ]. Additionally, in many rural schools, there is a lack of structural support for staff training and resources to administer high-quality PA lessons [6, 8–10]. Thus, there are many barriers that children from rural, low-SES backgrounds face in terms of accessing PA and building healthy PA behaviors.

Rates of childhood obesity have been rising while simultaneously the required amount of physical education (PE) in elementary and middle schools has been falling [5, 11]. Approximately, only 8% of middle schools and 4% of elementary schools offer PA opportunities daily [12]. While more structured PA would be beneficial for children both in and out of school hours, the lack of resources makes this difficult. High-quality PE programs are instrumental in promoting immediate health benefits and in fostering motor skills, confidence, and enjoyment of PA [13]. These health benefits and skills are crucial for encouraging lifelong engagement in PA thereby supporting long-term health and well-being [14].​.

There are few PA programs that are feasible, effective, and sustainable in promoting PA in children. Feasibility data determines the effectiveness of the intervention and how to improve implementation to promote a maximal positive effect [15]. The present intervention, called Hoosier Sport, was created with input from rural middle school staff, students, and the research team to develop a sustainable model to promote PA in rural schools. The model trains college students as implementers of the interventions, an underutilized approach [16]. Hoosier Sport aims to increase PA by providing enhanced PE classes led by trained college student role models, exposure to sport-based curriculum, and sport equipment access [16–18]. By incorporating service-learning, Hoosier Sport not only promotes PA engagement among youth but also aims to cultivate a generation of professionals committed to community-based health promotion [16–18].

Thus, we conducted a 1-year prospective longitudinal study of children from a rural Midwestern middle school. The primary objective of this study was to evaluate intervention feasibility, particularly focusing on the trial- and intervention-related indicators. We hypothesized that the intervention will be feasible, acceptable, and appropriate, with high engagement and positive perceptions among participants. The secondary objective was to assess preliminary efficacy and outcome trends in accelerometer-collected PA data from pre-, mid-, to post-intervention, as well as changes in physical measures (i.e., maximum plank test, 6-minute walk test). We anticipated greater PA levels in the intervention group at mid- and post-intervention timepoints. The exploratory assessments examined relationships between PA, physical competence, and demographic factors (i.e., biological sex, grade).

## Methods

### Overall study design

**Fig. 1:**
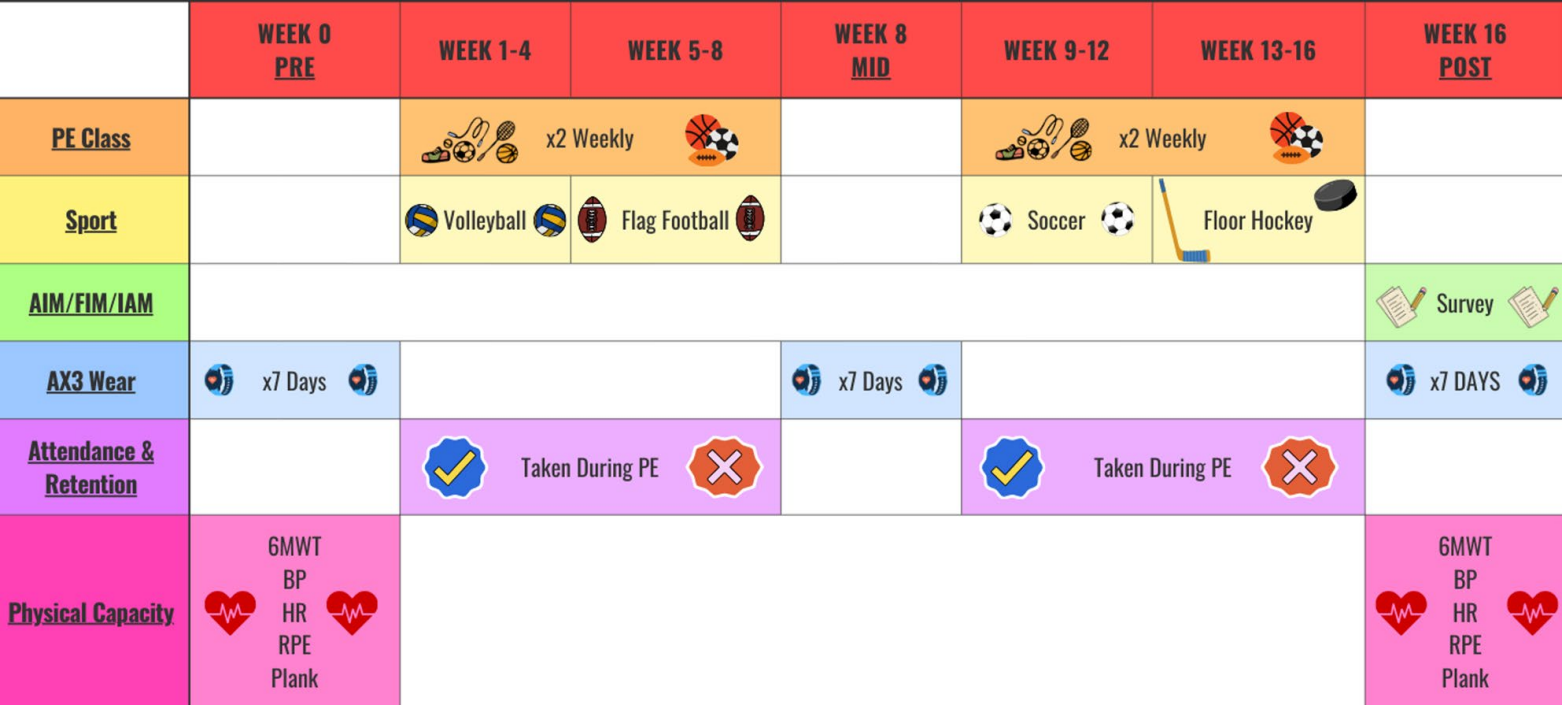
Data collection timepoints

This longitudinal cohort study and the data collection timeline are represented in Fig. 1. If students were enrolled in PE class, then they were assigned to the test condition (described below); if they were not enrolled in PE class, they were assigned to the control condition. The study took place over one academic year, with 8-weeks of intervention delivery in the fall semester and 8-weeks in the spring semester. There were pre-, mid-, and post-data collections with varying measures identified below.

### Conceptual framework

**Fig. 2:**
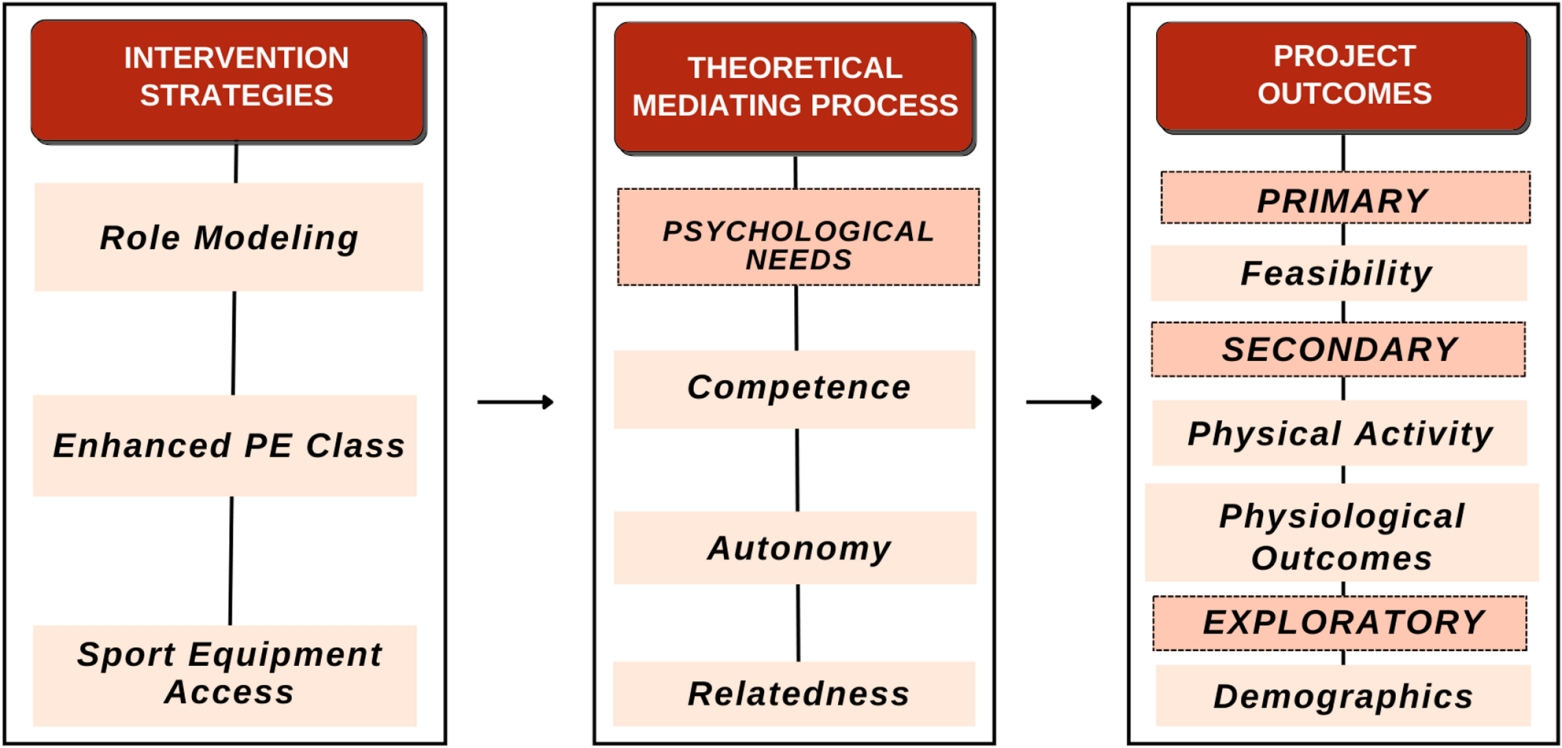
Conceptual framework

This intervention is based on the psychological needs mini-theory, which is a part of the self-determinant theory (SDT) [19]. The psychological needs mini-theory expresses humans have three psychological needs – autonomy, competence, and relatedness – that are necessary to maintain one’s intrinsic motivation and wellbeing [20, 21]. The psychological needs mini-theory is not in depth within this paper, to ensure main focus is on PA, however, psychological needs is mentioned more in partnering papers. The aim is to respectively focus on the PE class, sports played, feasibility (FIM, AIM, and IAM), and AX3 wear. We tracked the number of days or weeks needed for our focused targets to recognize feasibility and understand results. SDT shapes the determinants observed and tested to address the effectiveness of the program in minimizing CVD risk later in life. The effectiveness of a program is required to indicate resources are utilized wisely to achieve the desired outcome and necessary changes can be made. See Fig. 2 for the conceptual framework.

### Setting & sample

The study sample consisted of children from a rural, economically lower resourced community in the Midwestern United States. The study primarily took place during physical education classes and involved recruiting 6th, 7th, and 8th grade students from a rural middle school. The study included 83 participants, with 41 females and 42 males, grades 6th to 8th. All research components were carried out within a school setting, with the intervention taking place during physical education (PE) class periods. The Indiana University Institutional Review Board approved the study protocol (#18784).

#### Test condition (i.e., Hoosier Sport)

Hoosier Sport is a sport-based youth development program delivered by a Midwestern university. Hoosier Sport establishes community partnerships to deliver sport-based PA interventions in rural middle schools. The interventions include various sport curricula, college student role modeling, sport equipment access, and life skill development. More specifically, Hoosier Sport was delivered twice per week during PE class across fall and spring semesters (8 weeks each semester). Each class was 44-minutes. Hoosier Sport introduced fundamental sport skills through 4-week units (volleyball, flag football, soccer, floor hockey) led by trained college student implementers. Repeated exposure within each sport was designed to foster competence and enjoyment. Compared with standard PE curricula, Hoosier Sport emphasizes small-group instruction, progressive skill building, and individualized feedback. Importantly, the implementing middle school does not employ a trained PE teacher, so the Hoosier Sport program with multiple college student implementers (3–5) in each class and detailed curricular planning was significantly different than traditional PE at the middle school.

College student implementors were recruited through an undergrad exercise science class offered at the Midwestern university. Students that joined the class worked through the K450 class curriculum and training for 6-weeks prior to the start of the intervention. A requirement of 20 coaching hours at the intervention was expected from the students for the duration of the semester. Once the intervention started, there were 3–5 college student instructors at each intervention with an assigned lead coach and assistant coaches for each intervention day.

#### Control condition

Participants assigned to the control condition were not enrolled in PE classes. Instead, they were enrolled in either band, art, project-based STEM (science, technology, engineering, math), or computers. These participants took part in data collection at the same timepoints as the test condition.

### Procedures

Students from a rural middle school were recruited through lunch-time flyers, parent emails, and back-to-school handouts. Interested families were contacted to schedule a follow-up call, during which study details were discussed and verbal consent was obtained. Parents then completed the Physical Activity Readiness Questionnaire (PAR-Q) for their child and submitted written consent via an online form [22]. Once consent was received, students were excused from class on the first day of the intervention for data collection. During this session, students completed a brief in-person survey to provide assent, followed by additional surveys and fitness assessments. All assessments were conducted in a controlled setting by trained research assistants to ensure accuracy and provide support throughout the process. Data collection occurred at pre-, mid-, and post-intervention. All measures were collected at pre- and post-time points, aside from the AX3 data which was also collected at midpoint.

The first physical assessment was the 6MWT, followed by a 5-minute rest period before participants proceeded to the max plank test. Participants in the plank test maintained a plank position for the maximum duration they could. Researchers provided guidance to correct form, warning participants if their hips dropped too low or were raised too high.

### Measures

#### Trial-related feasibility indicators

We assessed recruitment by looking at the number of students and the total percentage of students that signed up in comparison to the total number of students within the school. Retention included the number of students that remained in the whole study and participated in post-data collection.

#### Intervention-related feasibility indicators

Within the questionnaire provided to each student, we used three key measures that were previously published, to assess intervention feasibility: AIM (Acceptability of Intervention Measure), IAM (Intervention Appropriateness Measure), and FIM (Feasibility of Intervention Measure) [18]. AIM evaluates how acceptable the program is to participants; IAM assesses whether the intervention is suitable for the target population; and FIM examines how realistic and practical it is to implement the program in real-world settings. Each measure used a Likert scale to gauge responses by using indications ranging from “completely disagree” to “completely agree” [18]. For each of the measures, the scores were summarized and calculated through the mean, median, and standard deviation, with the success threshold being defined as a median above 16.

#### Physiological outcomes

Exercise tolerance was evaluated with the 6-minute walk test and heart rate measurements before and after each assessment (pre- and post-intervention) [23]. These evaluations were conducted at the beginning (week 0) and end (week 16) of the intervention. The 6MWT involved participants walking as far as possible, back and forth the length of a gym for six minutes, while a researcher counted the completed laps. Heart rate (HR) was measured pre- and post- 6MWT to examine physical exertion during the test. The 6MWT and HR responses were used to find preliminary signals and identify physiological changes. These tests were practical in the setting and more enjoyable than the standard pacer test and weight measurements amongst this age group. The tests aim to decrease HR response at post-testing with similar or increased distance traveled with the 6MWT. Muscular endurance was evaluated with the plank test. Participants were shown proper plank form and attempted to hold a plank for as long as possible, without losing proper form. Participants had their elbows beneath their shoulders, legs straight, and feet together so their body was a straight line from head to toe.

#### Physical activity

This study used Axivity AX3 accelerometers (Axivity Ltd., UK), which are lightweight, triaxial devices shown to be reliable for measuring sedentary behavior and PA in children [24]. The AX3 records movement across three axes at 100 Hz within a ± 8 g range and accurately detects various activity types. Devices were worn on the non-dominant wrist using adjustable wristbands. Participants received clear instructions, including visual cues, to ensure correct orientation. Accelerometers were initialized before use and set to begin recording the day after distribution to reduce reactivity. Participants were asked to maintain typical activity patterns during the 7-day monitoring period. The accelerometers tracked the intensity of physical activities and categorized the activity into light, moderate, and vigorous. The accelerometers monitor how long the participants are in each intensity zone and their total PA for each day.

#### Physical competence and daily behavior

The present study used two domains of the Canadian Assessment of Physical Literacy-2 (CAPL-2): physical competence and daily behavior [25]. Physical competence was assessed using the plank test. Once the participants felt comfortable with the positioning, they were timed to see how long they could hold the plank before losing proper form. Daily behavior was self-reported by the participants by asking them how many days a week they endure 60-minutes of PA. For participants under the age of 15, the CAPL-2 is considered highly reliable (α: 0.71–0.97) [26]. One section of the CAPL-2, focusing on self-reported moderate-to-vigorous activity (MVPA), was instrumental in determining the participants’ level of effort and work rate.

### Data analysis

All analyses were conducted using RStudio (Version 4.5.1). Descriptive statistics (means, standard deviations, medians, and ranges) were computed for all variables at each time point. To evaluate the effects of the intervention, both within-subject (pre-, mid-, and post-intervention) and between-group comparisons were performed based on the outcome measures. For variables collected at baseline and post-intervention only—including physiological measures (HR, 6MWT distance, plank duration)—linear mixed models were used to assess changes within-group and between-groups. For measures collected at all three time points (pre-, mid-, and post-intervention)—specifically the PA data captured by AX3 accelerometers—linear mixed models were conducted to assess changes over time as a robust method for handling missing data. PA intensity levels and duration were identified using summary reports provided by GGIR software. We classified PA intensity using Euclidean Norm Minus One (ENMO) cut-point criteria of 100 mg (light), 400 mg (moderate), and 700 mg (vigorous) [27]. Effect sizes (Cohen’s *dz*) were calculated and interpreted based on conventional thresholds. Descriptive statistics were used to summarize feasibility-related metrics, including recruitment and retention rates, and compliance. For child-reported feasibility measures (AIM, IAM, FIM), central tendencies (mean, median) and standard deviations were calculated, with a median score above 16 considered indicative of high feasibility. Missing data was assumed to be missing at random. Statistical significance was set at *p* < 0.05 for all analyses.

Given the repeated measures design at three time points (pre-, mid-, and post-intervention) and our primary interest in detecting within-subject changes over time as well as between-group differences, we focused our analysis on linear mixed models with within–between interaction effects. To appropriately determine the suitable sample size, we used G*Power to carry out power analyses [28]. We specified a moderate effect size (Cohen’s f = 0.25), an alpha level of 0.05, and a desired statistical power of 0.80. The power analysis indicated that a minimum of 54 participants would be required to detect statistically significant effects under these parameters. To ensure sufficient power while also accounting for potential attrition, we aimed to recruit at least 60 participants. This sample size was expected to provide robust power to detect meaningful changes across time points and between conditions.

## Results

### Demographics & descriptive data

A total of 83 participants were enrolled in the study, with 49% females and 51% males, including 6th -8th grade students. Demographics, physiological outcomes, and PA are presented in Table 1.

**Table 1:** Characteristics of the study sample by timepoint

	Pre	Mid	Post
Demographics			
Grade (*n*, %)			
6th	24 (30.4%)	-	-
7th	29 (36.7%)	-	-
8th	30 (32.9%)	-	-
Gender (*n*, %)			
Female	41 (49%)	-	-
Male	42 (51%)	-	-
Physiological Outcomes (mean (*SD*))			
Plank (seconds)	65.3 (49.0)	-	68.0 (52.4)
6MWT (ft/sec)	486 (93.9)	-	454 (58.7)
HR Before	76.8 (12.3)	-	85.9 (14.5)
HR After	85.2 (15.3)	-	90.1 (16.9)
Physical Activity (mean (*SD*))			
Light (mins)	203 (78.2)	159 (72.2)	153 (50.4)
Moderate (mins)	127 (59.5)	117 (51.9)	108 (45.1)
Vigorous (mins)	18.6 (19.9)	21.4 (16.5)	15.1 (13.4)
Total (mins)	349 (139)	297 (124)	275 (99.6)
Self-Reported PA	4.78 (1.8)	-	4.71 (1.8)

For categorical variables, data are presented as n, % of sample; for continuous variables, data are presented as mean (*SD*); *6MWT* 6-Minute Walk Test; *HR* Heart Rate, Self-reported PA in days per week

### Primary objective: feasibility results

#### Recruitment & retention

83 middle school students were recruited in the fall semester (control group = 50; test group = 33), for a total of 55.3% of the total middle school students. Of the 83 middle school students that were recruited, 68 (81.9%) were retained as measured by post-intervention survey completion.

#### Feasibility, acceptability, appropriateness

FIM, AIM, and IAM scores exceeded the success threshold (median > 16) when assessed at post-intervention. Descriptive results for feasibility, acceptability, and appropriateness are presented in Table 2.

**Table 2:** Descriptive results for FIM, AIM, and IAM at post-intervention

	Full Sample (*n* = 83)
FIM	
Mean (*SD*)	16.6 (3.16)
Median [Min, Max]	17.0 [4.00, 20.0]
Missing	15 (18.1%)
AIM	
Mean (*SD*)	16.9 (3.23)
Median [Min, Max]	18.0 [4.00, 20.0]
Missing	15 (18.1%)
IAM	
Mean (*SD*)	16.8 (3.27)
Median [Min, Max]	17.0 [4.00, 20.0]
Missing	15 (18.1%)

### Secondary objective: physical measures

#### Light PA

Overall, there was a significant decrease in the minutes of light PA from baseline to mid-point (unstandardized *β* = 180.10 (95% *CI*: 159.93, 206.25), *p* = 0.020). Within the control group, the change in the estimated light PA from baseline (*M* = 180.1, *SE* ± 13.2) to mid-point (140.7 ± 15.5) was marginally significant (*t*(71.48) = -2.37, *p* = 0.053, *dz* = -0.83). The change from baseline (222.85 ± 15.19) to mid-point (182.04 ± 16.76) was marginally significant for the intervention group (*t*(69.14) = -2.30, *p* = 0.063, *dz* = -0.859), while there was a significant change from baseline to post (165.25 ± 17.18) (*t*(58.27) = -3.39, *p* = 0.004, *dz* = -1.213).

**Fig. 3:**
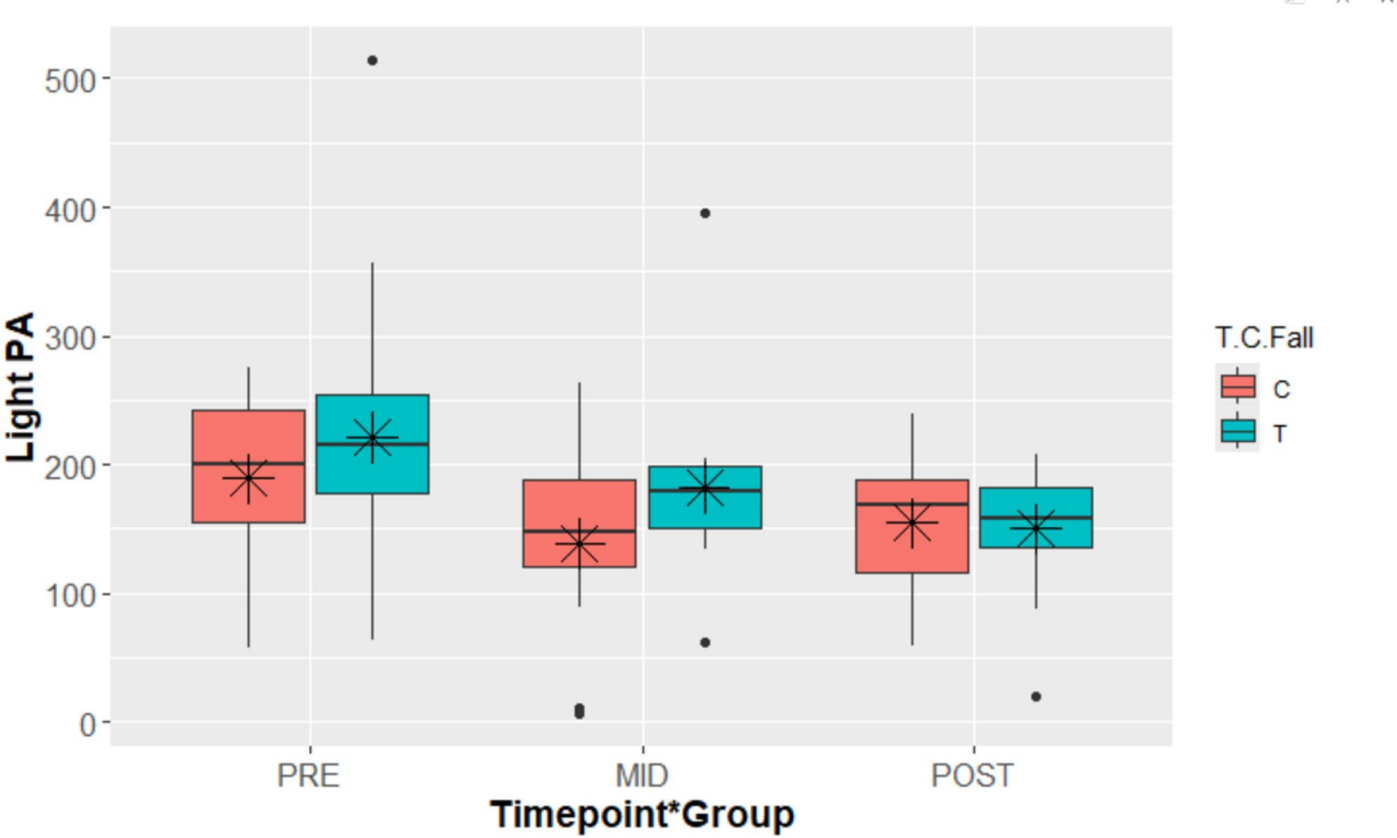
Boxplot of light PA by timepoint

For between-group comparisons, the intervention group had an estimated 42.76 (± 20.14) more minutes of light PA compared to the control group (*t*(101.02) = 2.12, *p* = 0.036, *dz* = 0.901) at baseline. The difference between the groups at mid-point was marginally significant (*t*(111.35) = 1.81, *p* = 0.073, *dz* = 0.871), with the intervention group exercising 41.37 min more of light PA than the control group. The estimated difference in minutes of light PA at post was 8.40 (*t*(110.64) = 0.38, *p* = 0.7, *dz* = 0.177). There were no significant differences in the changes of light PA over time between the groups (*p* > 0.05). See Fig. 3 for differences in PA by timepoint.

#### Moderate PA

The minutes of moderate PA decreased slightly from baseline to mid-point (*β* = -12.518, 95% *CI*(-40.371, 15.336)) and baseline to post (*β* = -12.998, 95% *CI*(-38.758, 12.761)), but neither of these differences were significant (*p* = 0.373 & 0.317, respectively). Within each group, none of the differences within timepoint were significant (*p* > 0.05), as well as the differences between groups within each timepoint (*p* > 0.05).

#### Vigorous PA

There was a slight increase in the minutes of vigorous PA from baseline to mid (*β* = 6.364, 95% *CI*(-2.236, 14.964)), but a decrease overall from baseline to post (*β* = -2.082, 95% *CI*(-10.022, 5.858)), but neither of these differences were significant (*p* = 0.145 & 0.603, respectively). The changes in time for vigorous PA were neither significant within-group or between-group (*p* > 0.05). There were no differences between groups at any of the timepoints (*p* > 0.05).

#### Total PA

Total minutes of PA decreased from baseline to mid (*β* = -42.762, 95% *CI*(-101.220, 15.696)) and baseline to post (*β* = -35.952, 95% *CI*(-89.617, 17.713)), but neither of these differences were significant (*p* = 0.149 & 0.185, respectively). Within the intervention group, there was a significant decrease in total minutes of PA from baseline (377.78 ± 26.89) to post (301.70 ± 30.41) (*t*(58.32) = -2.52, *p* = 0.038, *dz* = -0.903). No significant differences in change in timepoint were observed in the control group. At baseline, the intervention group had an estimated 64.91 more total minutes of PA compared to the control group (*t*(101.13) = 1.82, *p* = 0.072, *dz* = 0.771), but this difference is considered marginally significant. There were no significant differences in change over time of total PA between the groups (*p* > 0.05). Parameter estimates are shown in Table 3 for light, moderate, vigorous, and total PA.

**Table 3:** Parameter estimates for light, moderate, vigorous, and total PA

	Light PA			Moderate PA			Vigorous PA		
Characteristics	Beta	95% *CI*	*p*-value	Beta	95% *CI*	*p*-value	Beta	95% *CI*	*p*-value
(Intercept)	180.09	153.93, 206.25	0.000	119.88	99.71, 140.06	0.000	14.95	8.63, 21.26	0.000
Timepoint									
PRE	-	-	-	-	-	-	-	-	-
MID	-39.41	-72.45, -6.37	0.020	-12.52	-40.37, 15.34	0.373	6.36	-2.24, 14.96	0.145
POST	-23.23	-53.57, 7.11	0.131	-13.00	-38.76, 12.76	0.317	-2.08	-10.02, 5.86	0.603
T.C.Fall									
C	-	-	-	-	-	-	-	-	-
T	42.76	2.88, 82.64	0.036	11.37	-19.27, 42.02	0.463	7.87	-1.72, 17.47	0.107
Timepoint * T.C.Fall									
MID * T	-1.39	-49.77, 46.99	0.954	4.46	-36.42, 45.33	0.829	-9.22	-21.84, 3.39	0.150
Post * T	-34.36	-80.03, 11.31	0.137	-3.98	-43.04, 35.09	0.839	-1.20	-13.22, 10.82	0.843

*PA*  Physical activity, *CI*  Confidence Interval, *C*  Control group, *T*  Test group

### Other physical measures

#### Heart rate responses before and after 6-minute walk tests

Within the control group, at baseline there was a significant increase in HR after walking (84.94 ± 2.16) from before (77.73 ± 2.16) (*t*(205.44) = 3.25, *p* = 0.007, *dz* = 0.671), but the increase in HR before (87.46 ± 2.27) and after (90.38 ± 2.27) walking at post was not significant (*t*(205.44) = 1.23, *p* = 0.6, *dz* = 0.272). For the treatment group, baseline HR significantly increased from before (76.12 ± 2.64) to after (86.21 ± 2.64) walking (*t*(205.44) = 3.70, *p* = 0.002, *dz* = 0.939). In the post timepoint, HR did increase from before (84.58 ± 2.78) to after (90.76 ± 2.78) walking but this increase was not significant (*t*(205.44) = 2.11, *p* = 0.2, *dz* = 0.575). There were no significant differences in the HR before or after walking for 6 min between the groups at baseline and post.

**Fig. 4:**
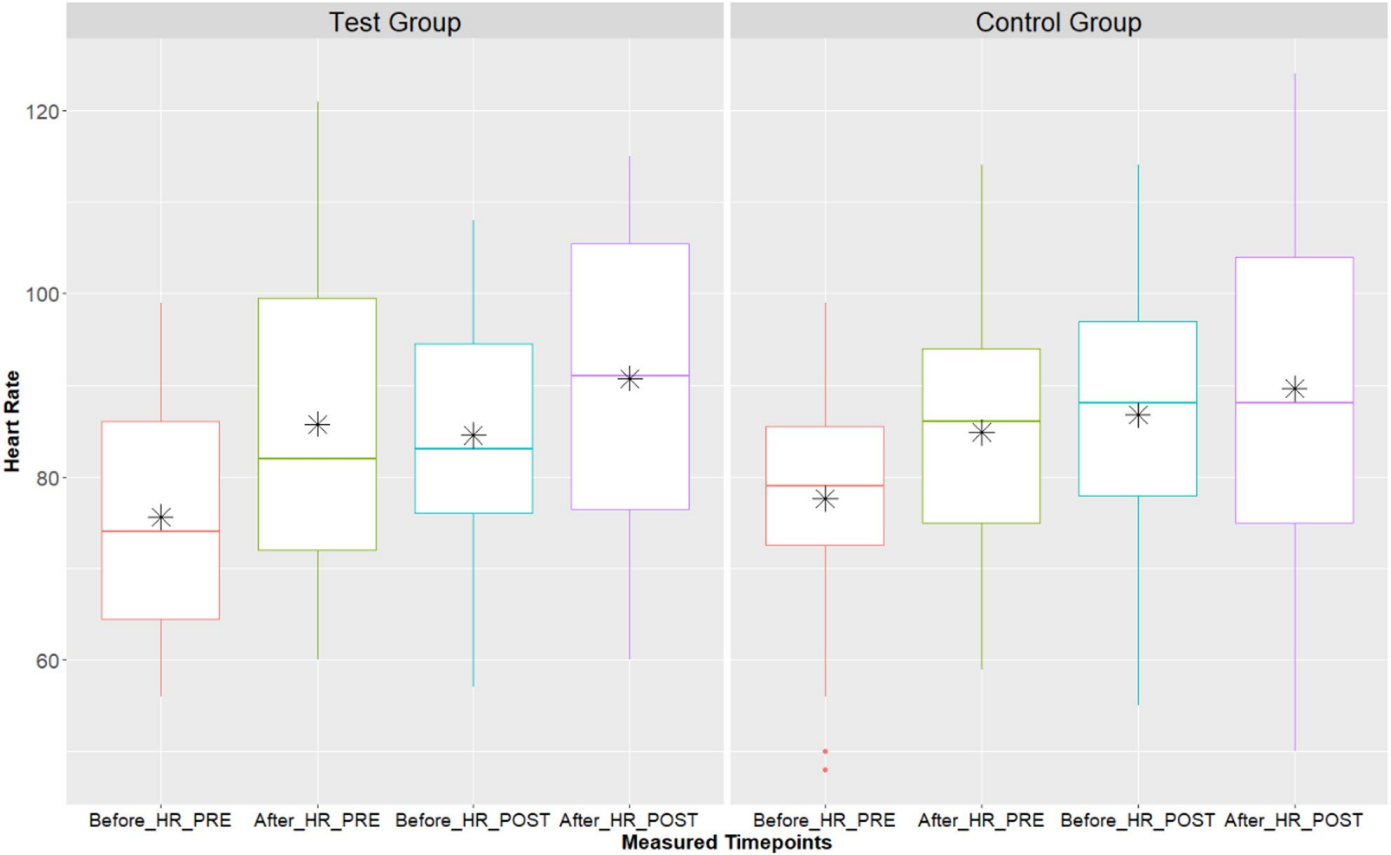
Boxplots of HR by timepoint

For change in HR rate (after walking HR – before walking HR), there was a significant decrease in the control group from post (2.96 ± 1.69) and baseline (7.24 ± 1.57) (*t*(71.89) = -2.03, *p* = 0.047, *dz* = -0.437). The intervention group showed a decrease in change in HR from baseline (10.00 ± 1.94) to post (6.24 ± 2.08), but this difference was not significant (*t*(75.60) = -1.44, *p* = 0.2, *dz* = -0.384). There were no significant differences in change in HR between the groups within each timepoint, nor was the change in HR over time significantly different between the groups (*p* > 0.05) as shown in Fig. 4.

#### Self-reported PA

**Fig. 5:**
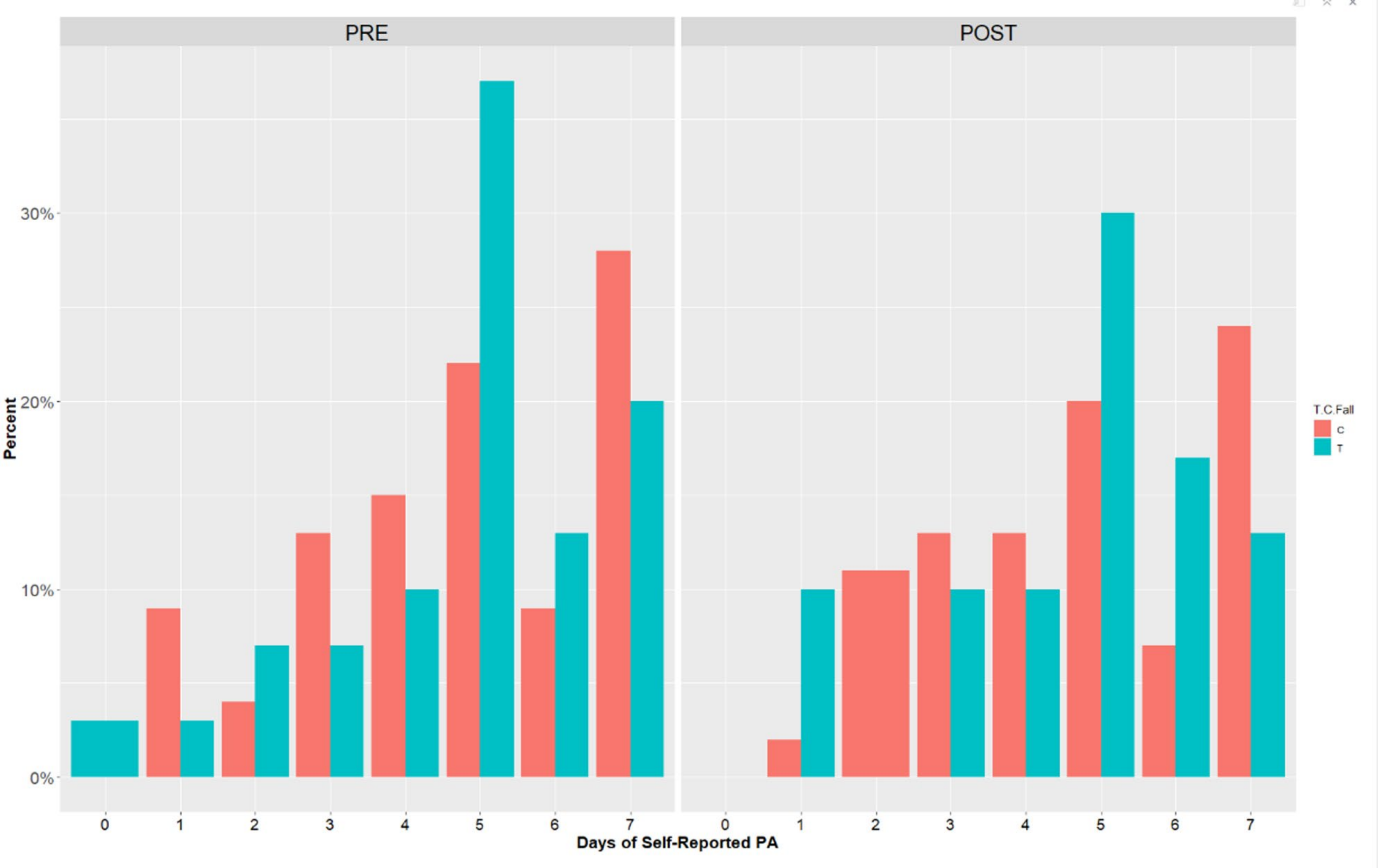
Self-reported PA by number of days at pre- and post-intervention

There were no significant differences between groups or timepoints for self-reported PA. See Table 4; Fig. 5 for self-reported PA results.

**Table 4:** Self-reported PA at pre- and post-intervention

	C (*n* = 50)	T (*n* = 33)	Overall (*n* = 83)
X60.in_PRE			
Mean (*SD*)	4.76 (1.92)	4.80 (1.83)	4.78 (1.87)
Median [Min, Max]	5.00 [1.00, 7.00]	5.00 [0, 7.00]	5.00 [0, 7.00]
Missing	4 (8.0%)	3 (9.1%)	7 (8.4%)
X60.Min_POST			
Mean (*SD*)	4.71 (1.83)	4.70 (1.77)	4.71 (1.80)
Median [Min, Max]	5.00 [1.00, 7.00]	5.00 [1.00, 7.00]	5.00 [1.00, 7.00]
Missing	9 (18.0%)	6 (18.2%)	15 (18.1%)

#### Maximum plank duration

There were no significant differences in plank duration between pre- and post-intervention timepoints (post *β* = -3.004, 95% *CI*(-12.603, 6.595)).

##### Exploratory objective

For the exploratory objective there were no significant correlations between accelerometer-derived PA, physical measures (i.e., plank, heart rate responses to 6MWT), and demographic factors (i.e., biological sex, grade).

## Discussion

The objective of the present study was to assess the feasibility of a 1-year sport-based PA intervention delivered by trained college students in a rural middle school. The study had 4 key findings. First, feasibility indicators exceeded a priori defined success threshold. Second, there were no significant differences in change over time of total PA between the test and control groups. Third, PA and other physical assessment results highlighted important opportunities for refinement to target improvements in 24-hour movement behaviors. Fourth, while community partner engagement and feasibility were high, opportunities for refinement and optimization in research design and methodology are needed. Overall, the present study was a major step forward within the iterative intervention lifecycle, moving towards more rigorous randomized controlled clinical trials. The present study highlights important community-based research implications and an informative example of a pragmatic PA intervention in rural schools.

The first key finding showed high trial- and intervention-related feasibility indicators (i.e., retention, recruitment, FIM, AIM, IAM). Successful recruitment and retention are likely due to having strong community relationships with the school and the children’s enjoyment of the intervention. Additionally, the college student implementers fostered an inclusive and energetic environment with the students. These findings align with previous literature showing high success rates when there is a strong relationship and built trust with community partners [29], as well as the potential value of college student (“near peer”) role models [30, 31]. Our model, which includes recruiting high-performing college students and training them for 6-weeks prior to intervention implementation, is entering its third year of existence – underscoring promising sustainability. Students earn academic course credits for completing the undergraduate training course which concludes with 8–10 weeks of service-learning opportunities (i.e., intervention delivery). College students then have opportunities for additional academic credits through enrollment in research-focused independent study. Hoosier Sport provides opportunities that rural communities may not have the resources for, specifically the ability to explore new sports curriculum and exposure to trained college student personnel and role models.

The second key finding was that there were no significant differences in change over time of light, moderate, vigorous, or total PA between the test and control groups. While there were some differences between PA at timepoints (e.g., light PA was lower at midpoint and post compared to baseline), the changes were not present when comparing the differences in change over time between groups. Many short-term children’s PA studies have demonstrated changes pre- to post-intervention [32, 33], but less are able to show sustained changes over time [34]. Changes are inconsistent due to the unknown level of activity and participation of students [33], the lack of comprehension for self-reported PA [34], and the delivery of interventions in a school setting compared to a remote setting [32]. Importantly, changes in PA was a secondary objective within the present study because the primary focus was on developing a sustainable infrastructure and competence to deliver larger-scale PA interventions. Although changes in PA were the secondary objective, this finding informs and strengthens our implementation strategies, enabling more effective delivery of large-scale PA interventions in the future.

The third key finding was that there are opportunities for refinement when observing 24-hour PA. The tests performed throughout the intervention such as the plank test, 6MWT, and HR monitoring provide excellent data for a momentary duration of time. At scale, the tests do not examine the student’s complete daily PA levels, so they do not explore total PA but instead endurance. The plank test, HR responses, and 6MWT identify physiological effects and are more practical than the standard pacer test and weight training amongst middle school students. The AX3 accelerometer data, however, presented us with data that explored full 24-hour PA levels. The AX3 data was nonsignificant, showing low compliance with students wearing the accelerometers due to extracurricular activities or forgetting to wear them. Relatedly, an important takeaway within this key finding is that future studies need to refine and improve the incentive system for reducing accelerometer data missingness. Accelerometer data missingness is a common challenge in children’s PA research and could be due to multiple factors including attrition and non-compliance [35, 36]. Traditionally, data missingness has been estimated as a rule of thumb at 20% attrition, and a recent review of children’s PA studies found an average missingness of 37% [35]. Our accelerometer data missingness of 42.2% (pre), 59.0% (mid), and 55.4% (post) are similar to other children’s PA studies using accelerometers but in need of improvement. While not always reported, researchers have tried different strategies to reduce missingness, including monetary compensation, reminder phone calls, SMS messages, and in-person visits. The present study offered $20 monetary compensation, but we want to increase that amount and/or scaffold the approach with another behavioral strategy. To increase compliance with AX3 wear, researchers suggest having a dual reward system for return of the accelerometer and the adherence to wearing the accelerometers [37].

The fourth key finding was that there are opportunities for refinement of research design and methodology in community settings. While the strong relationship with the school partner and administration supported high levels of feasibility and collaboration, several challenges emerged that affected the results. Issues of control group contamination arose, which is common in school-based interventions where students share environments and interact frequently. These challenges limited the ability to isolate intervention effects but also provided important lessons for improving trial design in subsequent trials. Despite these factors, the high engagement from school partners offered valuable insight into feasible solutions, including clearer communication of group roles, staggered implementation, or cluster-randomized designs. Future trials will include additional rural schools to delineate clearer test, control, and/or comparison groups. These refinements will help balance methodological rigor with the realities of community-based research and strengthen the design of future randomized controlled trials [38].

Despite the valuable contributions of this study, several limitations warrant consideration. First, the sample lacked demographic diversity, consisting predominantly of white students, which limits the generalizability of the findings. Second, due to constraints in the school’s existing class structure, the test and control groups were not randomized. This led to limited internal validity, as some participants engaged in extracurricular sports that could have influenced the outcome measures. Third, the sample size, while modest at 83 students, represented over 50% of the school’s total enrollment and was therefore a relative strength, despite increasing variability between groups. Fourth, while the study was originally designed as a crossover, inconsistent implementation during the spring semester prevented a full crossover analysis. Accordingly, analyses focused on initial test/control assignment. Fifth, the intervention was only delivered twice a week during PE classes (44 min per session), which may not be enough to see changes; however, implementation was coordinated in advance with school administrators for what was feasible. Lastly, effectiveness of the intervention may vary when incorporated to different schools. Despite these limitations, the intervention delivery using a sustainable pipeline of college students was a promising example of connecting a university to a local rural community for mutual benefit. Additionally, future studies should consider randomized designs when possible, expand participant diversity, and increase both the sample size and intervention duration to better support physiological outcomes and enhance internal validity.

## Conclusion

This study emphasizes the feasibility of a sport-based PA intervention in a relatively remote rural middle school setting. The intervention successfully met key indicators for feasibility, supporting its viability based on student perspectives, recruitment, and retention. Comparisons between test and control group were non-significant, but our research team’s competency in deploying accelerometers to rural children and delivering PA interventions in rural settings were increased. Notably, there are opportunities for refinement when evaluating PA levels, especially improving incentive systems to increase accelerometer compliance. Coupled with improving incentive systems, this research will be advanced in the future by adding additional rigor to the study design. For example, by including a second rural middle school, the research team will be able to isolate the control group from the test group and add an element of randomization for group assignment. Despite limitations, this study provides a strong foundation for refining school-based interventions aimed at improving PA levels.

## Data Availability

The datasets used and/or analyzed in the current study are available from the corresponding author on reasonable request.

## Abbreviations

PA: Physical Activity
PE: Physical Education
HR: Heart Rate
6MWT: Six Minute Walk Test
CVD: Cardiovascular Disease
SES: Socioeconomic Status
SDT: Self Determinant Theory
FIM: Feasibility of Intervention Measure
IAM: Intervention Appropriateness Measure
AIM: Acceptability of Intervention Measures
PAR-Q: Physical Activity Readiness Questionnaire
CAPL-2: Canadian Assessment of Physical Literacy
CI: Confidence Interval
SD: Standard Deviation
M: Mean
